# Synthesis of biogenic gold nano conjugate for increased efficacy and sustained drug release profile of saquinavir

**DOI:** 10.1186/1471-2334-14-S3-E41

**Published:** 2014-05-27

**Authors:** Rohan Kesarkar, Sweta Kothari, Abhay Chowdhary

**Affiliations:** 1 Department of Virology and Immunology, Haffkine Institute for Training Research and Testing, Mumbai- 400012, India

## Background

Conventional anti retroviral treatment (ART) has been a core treatment for HIV over the past three decades. Despite the effectiveness of these drugs, most of them abide significant drawbacks such as poor bioavailability and undesirable side effects; thus reducing patient compliance and rendering treatment regime ineffective. This research work is an attempt to develop a dual pronged carrier system using gold nanoparticles (GNP) for increasing effectiveness and sustained drug release of saquinavir.

## Methods

Leaf extract of *Azadirachta indica* was used as an efficient sink for synthesis of GNPs by microwave assistance. Saquinavir (Saq) was attached onto GNPs that were pre functionalized by capping polypeptide linkers of *A.indica* and characterized by FTIR. Percentage drug loading efficiency (%DLE) and release kinetics were studied by statistical model dependent method. Biocompatibility of drug nanoparticle conjugate was studied by MTT assay. Antiviral efficacy was assessed by quantifying aspartic protease of HIV by fluorescence (FRET) method.

## Results

Mono dispersed GNPs (30-40 nm) were synthesized and found to carry high payloads of saquinavir with %DLE of 93.6% as analyzed by Transmission electron microscopy and UV Vis spectroscopy respectively. GNP-saq conjugate demonstrated 16.7% increase in anti viral efficacy as compared to saquinavir alone. The conjugate showed sustained drug release and followed 1st order release kinetics at physiological pH (7.2).

## Conclusion

Biogenic GNPs serve as a potent candidate for ferrying high payloads of anti retroviral drugs with increased efficacy and reduced side effects. This approach can help reduce dosage frequency and improvise treatment strategy against HIV.

